# Statistically optimal estimation of Greenland Ice Sheet mass variations from GRACE monthly solutions using an improved mascon approach

**DOI:** 10.1007/s00190-017-1063-5

**Published:** 2017-09-13

**Authors:** J. Ran, P. Ditmar, R. Klees, H. H. Farahani

**Affiliations:** Delft University of Technology, Stevinweg 1, 2628 CN Delft, The Netherlands

**Keywords:** GRACE, Mascon, Data weighting, Greenland Ice Sheet, Surface mass balance

## Abstract

We present an improved mascon approach to transform monthly spherical harmonic solutions based on GRACE satellite data into mass anomaly estimates in Greenland. The GRACE-based spherical harmonic coefficients are used to synthesize gravity anomalies at satellite altitude, which are then inverted into mass anomalies per mascon. The limited spectral content of the gravity anomalies is properly accounted for by applying a low-pass filter as part of the inversion procedure to make the functional model spectrally consistent with the data. The full error covariance matrices of the monthly GRACE solutions are properly propagated using the law of covariance propagation. Using numerical experiments, we demonstrate the importance of a proper data weighting and of the spectral consistency between functional model and data. The developed methodology is applied to process real GRACE level-2 data (CSR RL05). The obtained mass anomaly estimates are integrated over five drainage systems, as well as over entire Greenland. We find that the statistically optimal data weighting reduces random noise by 35–69%, depending on the drainage system. The obtained mass anomaly time-series are de-trended to eliminate the contribution of ice discharge and are compared with de-trended surface mass balance (SMB) time-series computed with the Regional Atmospheric Climate Model (RACMO 2.3). We show that when using a statistically optimal data weighting in GRACE data processing, the discrepancies between GRACE-based estimates of SMB and modelled SMB are reduced by 24–47%.

## Introduction

One of the primary sources of information about mass variations of the Greenland Ice Sheet (GrIS) is the Gravity Recovery and Climate Experiment (GRACE) satellite mission. Using primarily K-band ranging (KBR) data between the two GRACE satellites, monthly sets of spherical harmonic coefficients (SHCs) are computed complete to some maximum degree, e.g. 96 for CSR (the Center of Space Research of the University of Texas at Austin) RL05 solutions (Bettadpur 2012) and 90 for GFZ (GeoForschungsZentrum) RL05 solutions (Dahle et al. 2012). Alternatively, gravity solutions in terms of mass anomalies per mass concentration block (“mascon”) have also been released by Jet Propulsion Laboratory (JPL) (Watkins et al. 2015; Wiese 2015; Wiese et al. 2016), Goddard Space Flight Center (GSFC) (Luthcke et al. 2013) and CSR (Save et al. 2016). To clean KBR data from the contribution of high-frequency mass variations, an ocean tide model [e.g. EOT11a (Savcenko and Bosch 2010)], a model of non-tidal components of the atmospheric and oceanic mass variations [e.g. the Atmosphere and Ocean De-aliasing model (AOD) (Dobslaw et al. 2013)] and other background models are routinely used.

The sensitivity of GRACE measurements is known to be anisotropic: it is higher in the along-track direction and lower in the cross-track direction (Condi et al. 2004; Ditmar et al. 2012). A higher sensitivity amplifies data errors, which are caused, among others, by noise in the data provided by on-board sensors and imperfectness of background models. As a result, monthly sets of SHCs are contaminated by strong north–south “stripes”, with amplitudes that depend on the latitude (Wahr et al. 2006). These amplitudes are smaller in polar areas and larger near the equator (Wahr et al. 2006; Linage et al. 2009).

In principle, gravity field variations expressed in spherical harmonics can be converted into mass anomalies at the Earth’s surface by a spectral transfer using a proper scaling of SHCs (Wahr et al. 1998). To suppress stripes and high-frequency noise, low-pass filters and/or de-striping schemes are typically used (Jekeli 1981; Wahr et al. 1998; Swenson and Wahr 2006) at a price of a reduced spatial resolution and distortions in the estimated mass anomalies (Duan et al. 2009).

Alternatively, the mass anomalies can be estimated from the SHCs using least-squares techniques. In this case, they are modelled as a thin mass layer located at the Earth’s surface, or some approximation of it. The mass layer is introduced as a constant function over mascons of pre-defined geometries. The geometry of the mascons can be chosen to take into account existing physical constraints, like the geometry of the coastal line. A proper choice of the size of the mascons allows for noise suppression without the need for any additional filtering of the SHCs, e.g. de-striping scheme. This helps in reducing distortions in the estimated mass anomalies. Luthcke et al. (2006) were the first to use the mascon representation to derive mass anomalies over Greenland from GRACE level-1b data, followed by Luthcke et al. (2013), Watkins et al. (2015) and Save et al. (2016). To reduce the numerical complexity, variants of the mascon approach have been suggested, which use monthly sets of SHCs as input, e.g. Forsberg and Reeh (2007), Baur and Sneeuw (2011) and Schrama and Wouters (2011). In line with Forsberg and Reeh (2007) and Baur and Sneeuw (2011), we compute monthly sets of gravity disturbances at a mean satellite altitude from the monthly sets of SHCs as data to estimate mass anomalies per mascon.

The major objective of the present study is to develop a statistically optimal variant of the mascon approach applicable to the estimation of Greenland mass anomalies. We suggest a number of improvements upon Forsberg and Reeh (2007) and Baur and Sneeuw (2011). Two of the major improvements are described here. Firstly, we properly propagate the full error covariance matrices of monthly SHCs into gravity disturbances at satellite altitude using the law of covariance propagation. These noise covariance matrices of gravity disturbances are used in the subsequent least-squares adjustment. We expect a noticeable improvement in the estimated mass anomalies and their uncertainties, as noise in SHCs is highly correlated (Swenson and Wahr 2006), among others due to the anisotropic sensitivity of the GRACE KBR data. To address the ill-conditioning of the propagated noise covariance matrices, we develop an approximate inversion scheme based on an eigenvalue decomposition. Secondly, we ensure a spectral consistency between the GRACE-based gravity disturbances and the unknown mascon parameters. The spectrum of the GRACE-based gravity disturbances is limited by the maximum spherical harmonic degree of the monthly sets of SHCs, whereas the mascon representation implies that gravity disturbances contain energy at higher frequencies, too. The spectral consistency has not been considered in previous studies, which is partially due to the fact that in these studies scaled unit matrices were used to represent the data noise. When using full noise covariance matrices as in this study, spectral consistency between model and data noise is indispensable to obtain high-quality solutions.

Typically, the mascon approach makes use of regularization or other spatial constraints to suppress noise at a price of introducing a bias in the solution. In this study, no spatial constraints in the form of regularization are used. Instead, the size of the mascons is chosen carefully in order to control the noise.

To demonstrate the performance of the proposed methodology, we make use of both synthetic and real data. In the latter case, we exploit GRACE Release-05 monthly solutions provided by CSR. To investigate the importance of proper data weighting and for validation, we compare the estimated mass anomalies with surface mass balance (SMB) estimates from the Regional Atmospheric Climate Model (RACMO 2.3) (Noël et al. 2015). However, a direct comparison of GRACE-based and SMB-based mass anomalies is not possible because the latter time-series lacks the ice discharge signal. To solve that problem, we estimate and remove linear trends from both time-series. This is justified because seasonal mass variation signals of Greenland are dominated by SMB-related signals (van den Broeke et al. 2009).

The remaining part of the paper is organized as follows. In Sect. 2, we present the statistically optimal mascon approach. The performance of this approach is demonstrated using simulated data, which is the subject of Sect. 3. Particularly, we investigate to what extent the estimates are improved when incorporating the full noise covariance matrices and ensuring the spectral consistency between the data and the mascon parameters. In Sect. 4, we present the results of real data processing and validate them against SMB time-series. Finally, we provide a summary and the main conclusions in Sect. 5.

## Methodology

We propose an improved mascon approach compared to earlier studies by Forsberg and Reeh (2007) and Baur and Sneeuw (2011). Section 2.1 describes the exploited functional model, which is forced to be spectrally consistent with monthly GRACE SHCs. In Sect. 2.2, we discuss a practical way to divide the territory of Greenland into almost equal-area patches of irregular shape. The proper choice of the area over which gravity disturbances at satellite altitude are generated is discussed in Sect. 2.3. Section 2.4 describes the statistically optimal inversion of gravity disturbances into mass anomalies per mascon.

### Gravity disturbances

Monthly sets of gravity disturbances at mean satellite altitude are computed from monthly GRACE SHCs using spherical harmonic synthesis. Then, they are linked to the gravitational attraction of mascons at the Earth’s surface. Finally, mascon parameters are estimated using least-squares techniques.

#### GRACE-based gravity disturbances

In the context of this study, a gravity disturbance $\delta g$ is understood as the negative radial derivative of the gravitational potential *V*, generated by a mass anomaly:

1 $$\begin{aligned} \delta g = -\frac{\partial V}{\partial r}. \end{aligned}$$

They are linked to a set of GRACE SHCs ${\varDelta } C_{lm}$ and ${\varDelta } S_{lm}$ complete to degree *L* as

2 $$\begin{aligned} \delta g_p&=\frac{GM}{r_p^2} \sum _{l=1}^{L}\frac{l+1}{1+k'_l}\left( \frac{a}{r_p}\right) ^l\sum _{m=0}^{l} \bar{P}_{lm} \nonumber \\&\quad \times \,\left( \sin \phi _p) ({\varDelta } C_{lm} \cos m\lambda _p+ {\varDelta } S_{lm}\sin m\lambda _p\right) , \end{aligned}$$

where *GM* is the geocentric constant; *a* is the semi-major axis of the reference ellipsoid; $(r_p,\phi _p,\lambda _p)$ are spherical coordinates of a data point *p*, which in this study is assumed to be located at an altitude of 500 km above a mean Earth sphere; L is the maximum degree of the monthly GRACE solutions; and $\bar{P}_{lm}$ is the normalized associated Legendre function of degree *l* and order *m*. Notice that the expression contains the load Love numbers $k'_l$, which are introduced to eliminate the effects of the elastic response of the Earth to a load, which is included in the SHCs. The lateral distribution of data points is discussed in Sect. 2.3.

#### Gravity disturbances generated by a set of mascons

Suppose we have *N* mascons $M_i \ (i=1,2,\ldots ,N)$. The surface density (mass per unit area) of mascon *i* is denoted as $\rho _i$. Then, Eq. (1) can be rewritten as

3 $$\begin{aligned} \delta g_p = -\frac{\partial }{\partial r} \left( G \sum _{i=1}^{N} \rho _i \int _{M_i} \frac{\mathrm{d}s}{l_p} \right) = -\frac{\partial }{\partial r} \left( {G \sum _{i=1}^{N} \rho _i I_{i,p}} \right) , \end{aligned}$$

where *G* is the universal gravitational constant and

4 $$\begin{aligned} I_{i,p}=\int _{M_i} \frac{\mathrm{d}s}{l_p} \end{aligned}$$

with $l_p$ being the distance between an integration point and the data point *p*.

$I_{i,p}$ has to be computed using numerical integration. Here, we use a composed Newton–Cotes formula. The nodes are located on a Fibonacci grid (González 2010). The number of nodes of mascon *i* is denoted $K_i$. Then,

5 $$\begin{aligned} I_{i,p} \approx \sum _{j=1}^{K_i} w_{ij} \frac{1}{l_{ij,p}}, \end{aligned}$$

where $w_{ij}=S_i/K_i$ with $S_i$ the surface area of mascon *i*. The distance $l_{ij,p}$ between a Fibonacci point (*i*, *j*) with spherical coordinates $(r_{ij},\; \phi _{ij},\; \lambda _{ij})$ and the data point *p* with spherical coordinates $(r_p,\; \phi _p,\; \lambda _p)$ can be computed as

6 $$\begin{aligned} l_{ij,p}=(r_{ij}^2+r_p^2-2r_{ij} r_p \cos {\varPsi }_{ij,p})^{\frac{1}{2}}, \end{aligned}$$

where $$\cos {\varPsi }_{ij,p}=\sin \phi _p \sin \phi _{ij}+\cos \phi _p \cos \phi _{ij} \cos (\lambda _p-\lambda _{ij})$$.

Then,

7 $$\begin{aligned} \delta g_p\approx & {} G \sum _{i=1}^{N} \rho _i \sum _{j=1}^{K_i} w_{ij} (r_{ij}^2+r_p^2-2r_{ij} r_p \cos {\varPsi }_{ij,p})^{-\frac{3}{2}}\nonumber \\&\times \,(r_{ij}-r_p\cos {\varPsi }_{ij,p}). \end{aligned}$$

Equation (7) represents the functional model that relates the gravity disturbances and the surface densities of the mascons. In matrix-vector form, Eq. (7) can be written as

8 $$\begin{aligned} \mathbf {d} \approx \mathbf {A}'\mathbf {x}, \end{aligned}$$

where $\mathbf {x}$ is the vector of surface densities, $\mathbf {d}$ is the vector of gravity disturbances, and $\mathbf {A}'$ is the design matrix. The vector $\mathbf {x}$ is estimated from the vector of gravity disturbances $\mathbf {d}$ using weighted least-squares techniques.

The gravity disturbances of Eq. (2) have a limited bandwidth because the monthly GRACE solutions are limited to a certain maximum spherical harmonic degree. However, the gravity disturbances of Eq. (7) are not band-limited. Hence, the functional model, Eq. (8), is not correct as there is a spectral inconsistency between the data and the model. To obtain a spectrally consistent functional model, we need to apply a low-pass filter to the design matrix $\mathbf {A}'$, i.e. $\mathbf {A}'$ needs to be replaced by $\mathbf {A}$, where

9 $$\begin{aligned} \mathbf {A}=\mathbf {Y}\mathbf {A}', \end{aligned}$$

and $\mathbf {Y}$ represents the low-pass filter. Without such a low-pass filter, the short wavelengths of the estimated mascon solution would be biased towards zero.

To define a suitable low-pass filter, we need to remember that each column of the design matrix $\mathbf {A}'$ represents a set of gravity disturbances caused by a single mascon of unit surface density. Therefore, the filter operation can be implemented as follows. Firstly, gravity disturbances caused by a single mascon of unit surface density are computed on an equal-angular global grid. They are used as input to estimate a SH model of gravity disturbances complete to some maximum degree $L > L_G$ using spherical harmonic analysis. The SH model is truncated at the maximum degree $L_G$ of the monthly GRACE spherical harmonic models and successively used to synthesize a column of the design matrix $\mathbf {A}$, which corresponds to the single mascon. This procedure has to be followed for every mascon. The result is a design matrix $\mathbf {A}$, which is spectrally consistent with the information content in the data and the data noise covariance matrix.

The spectrally consistent analogue of Eq. (8) is written as

10 $$\begin{aligned} \mathbf {d}=\mathbf {A}\mathbf {x}+\mathbf {n}, \end{aligned}$$

where the vector $\mathbf {n}$ is introduced to account for noise in the GRACE-based gravity disturbances. This noise is assumed to be of zero mean and Gaussian. Furthermore, we assume that

11 $$\begin{aligned} D\{\mathbf {n}\}=\mathbf {C_d}, \end{aligned}$$

where $D\{\cdot \}$ is the dispersion operator and $\mathbf {C_d}$ is the data noise covariance matrix. The latter is computed on a month-by-month basis from the full noise covariance matrix of the monthly SHCs using the law of covariance propagation.

**Fig. 1:**
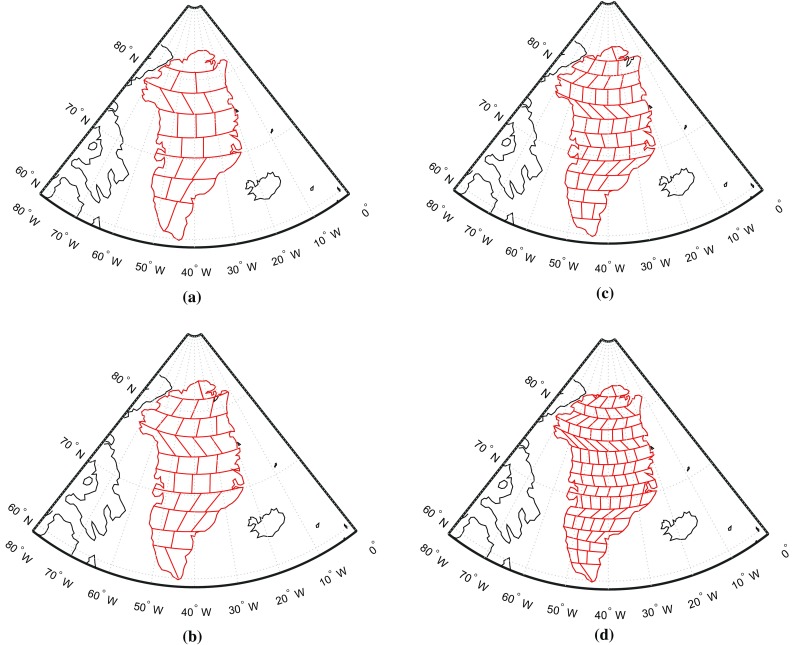
Partitioning of Greenland into 23 (size about $300 \times 300$ km), 36 (size about $250 \times 250$ km), 54 (size about $200 \times 200$ km) and 95 (size about $150 \times 150$ km) mascons, respectively

Then, best-linear unbiased estimator (BLUE) $\mathbf {\widehat{x}}$ of the mass anomalies is

12 $$\begin{aligned} \mathbf {\widehat{x}}=(\mathbf {A}^\mathrm{T} \mathbf {C_d}^{-1} \mathbf {A})^{-1}\mathbf {A}^\mathrm{T} \mathbf {C_d}^{-1}\mathbf {d}. \end{aligned}$$

The BLUE, Eq. 12, is referred to as the “statistically optimal estimator” in this study.

### Parameterization

The proper choice of the size of a mascon is important to mitigate noise amplification during the data inversion. To facilitate experiments with different mascon sizes, we developed a procedure for an automatic division of the territory of Greenland into nearly equal-area mascons of a desired size. The procedure consists of two steps. In the first step, Greenland is split into latitudinal strips of equal width, which is chosen to be as close to the desired size as possible. In the second step, each strip is split into individual mascons of an approximately desired size using straight segments in the rectangular projection. The orientation of the segments is adapted to follow the orientation of the west and east borders of the current strip. Examples of the resulting parameterizations are shown in Fig 1. Note that the mascons located at the Greenland coast are defined in line with the coastal geometry.

**Fig. 2:**
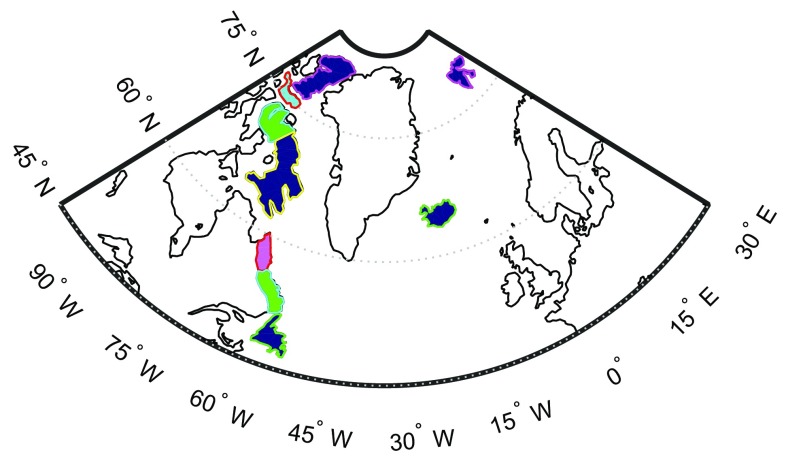
Mascons outside Greenland used in this study

We also define 9 mascons outside Greenland to reduce leakage of signal from outside Greenland into the Greenland mascons. These mascons cover Iceland, Svalbard and the Canadian Arctic Archipelago glaciers, see Fig. 2. It is worth mentioning that we do not parameterize the nearby ocean areas, due to a minor impact of oceanic mascons, e.g. at the level of 7 Gt/year for the trend over 2003–2013, when the optimal data weighting is applied.

### Distribution of data points

In choosing the altitude of data grid, we followed the suggestion of Baur and Sneeuw (2011): 500 km. Another option is to use altitudes between 480 and 500 km in order to address the decrease in orbital altitude of the GRACE satellites, as was done by Forsberg et al. (2017). Numerical studies (not shown here) reveal that this leads to similar estimates (around 10 Gt/year in terms of trend over 2003–2013) when the data weighting is switched on. We attribute the observed minor differences to the fact that the applied data processing strategy, including the truncation of the spectrum of the matrix Cd, was fine-tuned for the grid altitude of 500 km. We expect that fine-tuning of the data processing for grid altitudes chosen consistently with actual GRACE orbits would reduce these differences further. This was out of the scope of this study, but may be the subject of future research. The data area comprises Greenland and a buffer zone of 800 km around Greenland. The use of a buffer zone is justified by the fact that each gravity disturbance at satellite altitude is sensitive to a mass redistribution in a neighbourhood of a few hundred kilometres around that point (Baur and Sneeuw 2011). Thus, defining the data area in such a way ensures a more comprehensive representation of the target signals. The data points are located on a Fibonacci grid with a mean distance of 37.5 km. Additional data points on the oceans, but outside the data area are introduced for reasons discussed in Sect. 3.2.2. They are located on a Fibonacci grid with a mean distance of 2000 km. The total number of data points is 6953 with 6867 points inside the data area and 86 points in ocean areas outside the data area.

### Data inversion

The full noise covariance matrix of the GRACE-based gravity disturbances, $\mathbf {C_d}$, is ill-conditioned and possesses a gradually decreasing eigenvalue spectrum with many eigenvalues close to zero. Therefore, some kind of regularization is needed before this matrix is inverted. Here, we use an eigendecomposition to compute an approximate inverse, i.e.

13 $$\begin{aligned} \mathbf {C_d} = \mathbf {Q}\varvec{\Lambda } \mathbf {Q}^\mathrm{T}, \end{aligned}$$

where $\mathbf {Q}$ is a unitary matrix which contains the eigenvectors of $\mathbf {C_d}$ and $\varvec{\Lambda }$ is the square diagonal matrix of eigenvalues of $\mathbf {C_d}$. In Appendix A, we show that the matrices $\mathbf {Q}$ and $\varvec{\Lambda }$ can be computed without an explicit computation of the matrix $\mathbf {C_d}$, which helps to minimize the loss of significant digits.

**Table 1:** A summary of data used in this study

Data	Role	Temporal resolution	Spatial resolution	Pre-processing
ICESat elevation change rate	Simulating the true signal	2003–2009	20-km blocks	–
GRACE SHCs from DMT	Simulating signal leakage	Month	Degree 120	–
GRACE SHCs from CSR RL05	Real data	Month	Degree 96	–
Surface mass balance from RACMO 2.3	Validating estimates	Daily	11-km blocks	Resampled to monthly mean SMB for each drainage system and entire Greenland

Formally, the inversion of the matrix $\mathbf {C_d}$ can be written as

14 $$\begin{aligned} \mathbf {C_d}^{-1} = (\mathbf {Q}\varvec{\Lambda } \mathbf {Q}^\mathrm{T})^{-1} = \mathbf {Q}\varvec{\Lambda }^{-1} \mathbf {Q}^\mathrm{T}. \end{aligned}$$

However, many eigenvalues of the matrix $\mathbf {C}_d$ are small, reflecting the ill-conditioning of this matrix. Therefore, an approximate inverse of this matrix is computed as follows. The matrix $\varvec{\Lambda }$ is truncated in such a way that only the eigenvalues exceeding a pre-defined threshold are retained:

15 $$\begin{aligned} \varvec{\Lambda }_\mathbf{t} = \mathbf {J}\varvec{\Lambda }\mathbf {J}^\mathrm{T}, \end{aligned}$$

where $\mathbf {J}=[\mathbf {I} \ \ \mathbf {0}]$ is the truncation operator with $\mathbf {I}$ being a unit matrix and $\varvec{\Lambda }_\mathbf{t}$ is the resulting diagonal matrix, containing a truncated set of eigenvalues. By retaining only sufficiently large eigenvalues, we stabilize the computation of the inverse of the matrix $\varvec{\Lambda }_\mathbf{t}$. An approximate inverse $\tilde{\varvec{\Lambda }}^{-1}$ of the original matrix $\varvec{\Lambda }$ is obtained by replacing the missing elements with zeros:

16 $$\begin{aligned} \tilde{\varvec{\Lambda }}^{-1} = \mathbf {J}^\mathrm{T}\varvec{\Lambda }_\mathbf{t}^{-1}\mathbf {J}. \end{aligned}$$

After that, we define the approximate inverse ${\tilde{\mathbf {C}}}_{\mathbf{d}}^{-1}$ of the matrix $\mathbf {C_d}$ as

17 $$\begin{aligned} {\tilde{\mathbf {C}}}_{\mathbf{d}}^{-1} = \mathbf {Q}\tilde{\varvec{\Lambda }}^{-1}\mathbf {Q}^\mathrm{T} = \mathbf {Q}\mathbf {J}^\mathrm{T}\varvec{\Lambda }_t^{-1}\mathbf {J}\mathbf {Q}^\mathrm{T} = \mathbf {Q_t}\varvec{\Lambda }_t^{-1}\mathbf {Q_t}^\mathrm{T}, \end{aligned}$$

where

18 $$\begin{aligned} \mathbf {Q_t}=\mathbf {Q}\mathbf {J}^\mathrm{T} \end{aligned}$$

is the truncated matrix $\mathbf {Q}$ containing only the eigenvectors related to the retained eigenvalues. Then, according to Eq. (12), the weighted least-squares solution $\mathbf {\widehat{x}}$ is

19 $$\begin{aligned} \mathbf {\widehat{x}}&=(\mathbf {A}^\mathrm{T} {\tilde{\mathbf {C}}}_\mathbf{d}^{-1} \mathbf {A})^{-1}\mathbf {A}^\mathrm{T} {\tilde{\mathbf {C}}}_\mathbf{d}^{-1}\mathbf {d} \nonumber \\&=(\mathbf {A}^\mathrm{T} \mathbf {Q_t}\varvec{\Lambda }_\mathbf{t}^{-1} \mathbf {Q^\mathrm{T}_t} \mathbf {A})^{-1}\mathbf {A}^\mathrm{T} \mathbf {Q_t}\varvec{\Lambda }_\mathbf{t}^{-1}\mathbf {Q^\mathrm{T}_t}\mathbf {d} \nonumber \\&=(\mathbf {B}^\mathrm{T} \varvec{\Lambda }_\mathbf{t}^{-1} \mathbf {B})^{-1}\mathbf {B}^\mathrm{T} \varvec{\Lambda }_\mathbf{t}^{-1}\mathbf {Q^\mathrm{T}_t}\mathbf {d}, \end{aligned}$$

where

20 $$\begin{aligned} \mathbf {B}=\mathbf {Q^\mathrm{T}_t}\mathbf {A}. \end{aligned}$$

This solution is still unbiased, but strictly spoken not a minimum dispersion solution.

## Numerical experiments

We do a number of numerical experiments to investigate the performance of the improved mascon approach and to fine-tune some data processing parameters. In Sect. 3.1, we present the basic set-up of the numerical experiments. Section 3.2 is devoted to a presentation and discussion of the results. The importance of the spectral consistency is discussed in Sect. 3.3.

### Experimental set-up

The basic set-up used in all numerical experiments includes the definition of (i) the “true” signal and (ii) the error sources.

#### “True” signal

**Fig. 3:**
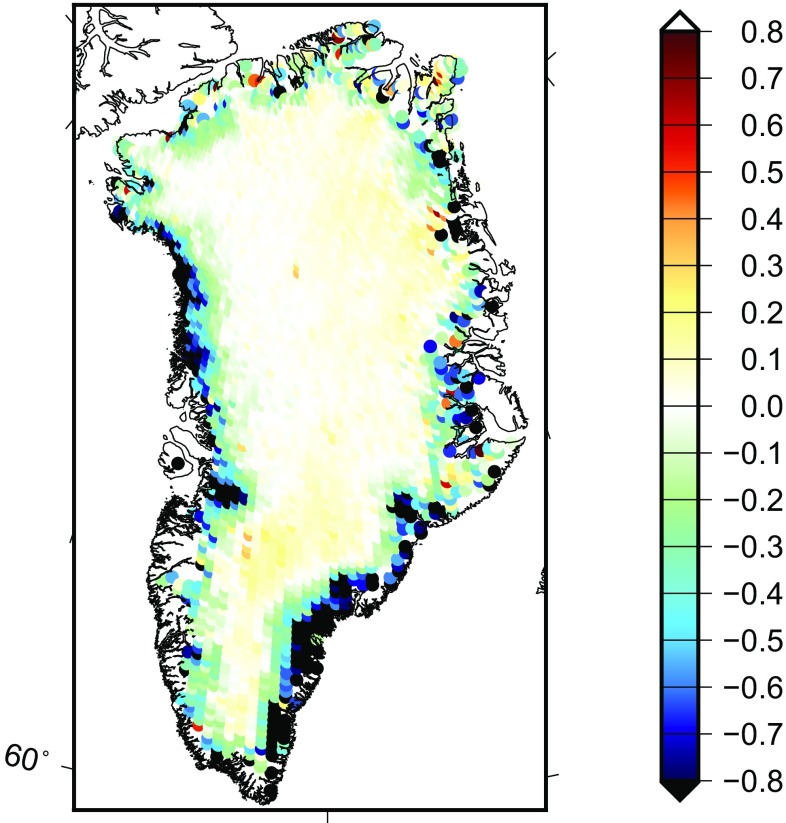
The “true” signal defined as the yearly mass change over the GrIS, in terms of EWH in units of metres

We define the “true” signal as the yearly mass change, which is determined on the basis of trends extracted from ICESat altimetry data (see Table 1) (Felikson et al. 2016). As shown in Fig. 3, these trends represent the mean rate of mass change over the period 2003–2009 per 20 $\times$ 20 km patch covering entire Greenland, converted from the surface elevation change rate by applying a density of 917 kg/$\text {km}^3$ (Wahr et al. 2000). This signal is directly used to compute the mass anomaly per mascon as “truth”. Using the proposed mascon approach, we generate gravity disturbances at satellite altitude from the ICESat altimetry data. Thereafter, we low-pass-filter them to limit the spectrum to spherical harmonic degrees from 1 to 120. Finally, we estimate mass anomaly per mascon and compare with the “truth” to evaluate the performance of the methodology.

**Fig. 4:**
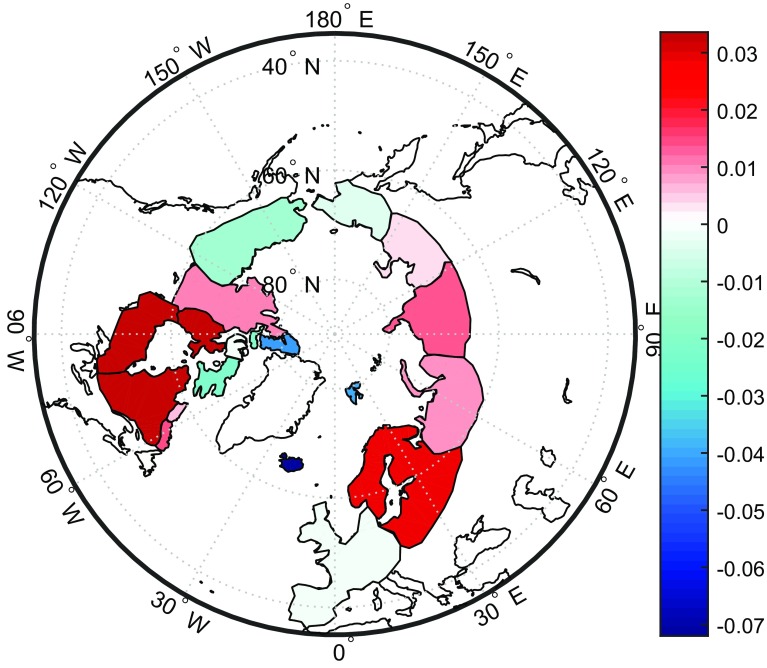
Mascons used to simulate signal leakage. The value of each mascon is the full signal generated using the trend over the period 2003–2008 derived from the DMT model, in terms of EWH in units of metres

There is much freedom in the definition of the “true” signal in the presence of secular trends. The “true” signal may reflect total mass change over an arbitrary time interval, ranging from one month to many years. The choice of the time interval determines the contribution of error sources like signal leakage and parameterization errors to the overall error budget. If the time interval is short (e.g. one month), signal leakage and parameterization errors may be small compared to the data noise. However, the relative contribution of these error sources to the overall error budget increases with increasing time interval. In this study, we define the “true” signal as the *yearly* mass change, which represents a kind of intermediate choice between the two extremes of a monthly signal and a multi-year signal. Our time interval is somewhat shorter than that considered in the study by Bonin and Chambers (2013), which was set equal to 4 years. In any case, the amplitude of the true signal in real GRACE data processing may differ depending on the signal of interest, which may range from short-term mass variations to long-term trends.

#### Error sources

The data generated in the previous section are superimposed by errors. In this study, we consider 4 error sources, i.e. signal leakage, AOD noise, random noise in GRACE-based SHCs and parameterization error. The latter is also sometimes referred to as “model error” (e.g. Xu 2010; Stedinger and Tasker 1986).

*3.1.2.1 Signal leakage* In this study, signal leakage refers to the impact of mass variations from outside Greenland on the estimated mascons. To simulate signal leakage, we introduce mass variations in Alaska, northern Canada, northern Russia and Fennoscandia, see Fig. 4. The “true” signal over these areas is also defined as the yearly mass variation. It is generated using the available optimally filtered trend over 2003–2008 based on the Delft Mass Transport (DMT) model (Siemes et al. 2013).

*3.1.2.2 AOD noise* AOD noise refers to errors in the background models, which are used to reduce non-tidal mass transport in the atmosphere and ocean. AOD error is considered to be one of large error sources in the monthly solutions. Here, we also take 10% of the difference of two AOD models separated by one year as the AOD noise, in line with the definition of the true signal (yearly mass accumulation). To that end, we choose AOD models in August of 2005 and 2006, because this period is roughly in the middle of the true signal (ICESat trend over 2003–2009). Based on our numerical study, we find that the AOD noise plays a minor role. Therefore, there would be negligible impact if a different time interval were chosen. Defining the AOD error as 10% of the AOD model signal is believed to be a reasonable choice, in view of previous studies (Thompson et al. 2004; Ditmar et al. 2012).

*3.1.2.3 Random noise* We assume that the yearly mass change is the result of the difference between two monthly solutions separated by a time interval of one year. Furthermore, we assume that there is no noise correlation between monthly solutions. This implies that the random noise in the generated yearly mass change can be set equal to the noise in a monthly solution multiplied with a factor of $\sqrt{2}$. First, we generate a vector $\mathbf {n}$ of zero-mean white Gaussian noise with unit variance; the length of $\mathbf {n}$ is equal to the number of SHCs. Then, a realization of correlated noise with the covariance structure of the matrix $C_{\delta _p}$ is obtained as

21 $$\begin{aligned} \mathbf {n_c}=\mathbf {L}\mathbf {n}, \end{aligned}$$

where $\mathbf {L}$ is the lower triangular Cholesky factor of the noise covariance matrix $\mathbf {C_{\delta _p}}$ of GRACE monthly SHCs:

22 $$\begin{aligned} \mathbf {C_{\delta _p}}=\mathbf {L}\mathbf {L}^\mathrm{T}. \end{aligned}$$

**Fig. 5:**
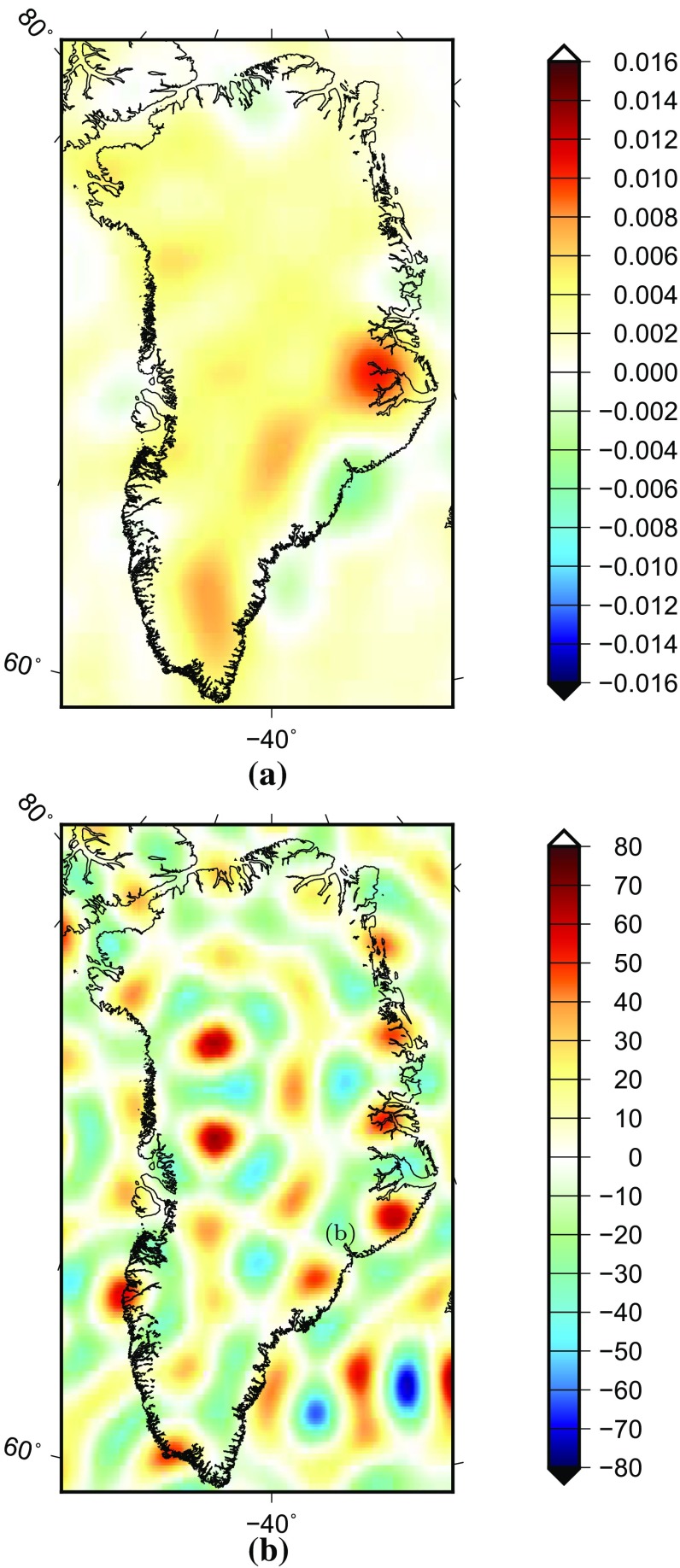
The *top panel* shows the AOD error, which is taken as 10% of the difference between August 2005 and August 2006. The *bottom panel* is a realization of simulated random errors based on the DMT noise covariance matrix of spherical harmonic coefficients for August 2006. (The matrix is complete up to degree 120.) The units are metres of EWH

In this study, the noise covariance matrix is complete to degree 120. It describes the noise in GRACE SHCs in August 2006 and was produced together with the DMT model. Note that the noise in the degree-one coefficients is not included. One hundred random noise realizations are simulated in this way in order to make the results of the numerical study more representative. Figure 5 shows one of these noise realizations in terms of EWH (equivalent water height).

*3.1.2.4 Parameterization errors* Parameterization errors are caused by the fact that the adopted parameterization assumes a uniform surface density distribution within each mascon, whereas the actual distribution within a mascon may spatially vary. Here, parameterization errors are automatically introduced, as the “true” signals are generated with ICESat altimetry data with a spatial resolution of 20 km, which is much finer than the mean size of a mascon.

**Table 2:** Optimal set of parameters for the estimation of total mass variations of entire Greenland

Options	Optimal choice
Width of the buffer zone around Greenland	800 km
Using additional data points over the global oceans	Yes
Number of mascons within Greenland	23
Optimal data weighting applied	Yes
Number of eigenvalues retained in the approximate inversion of $\mathbf {C_d}$	600
Spectral consistency maintained	Yes

**Fig. 6:**
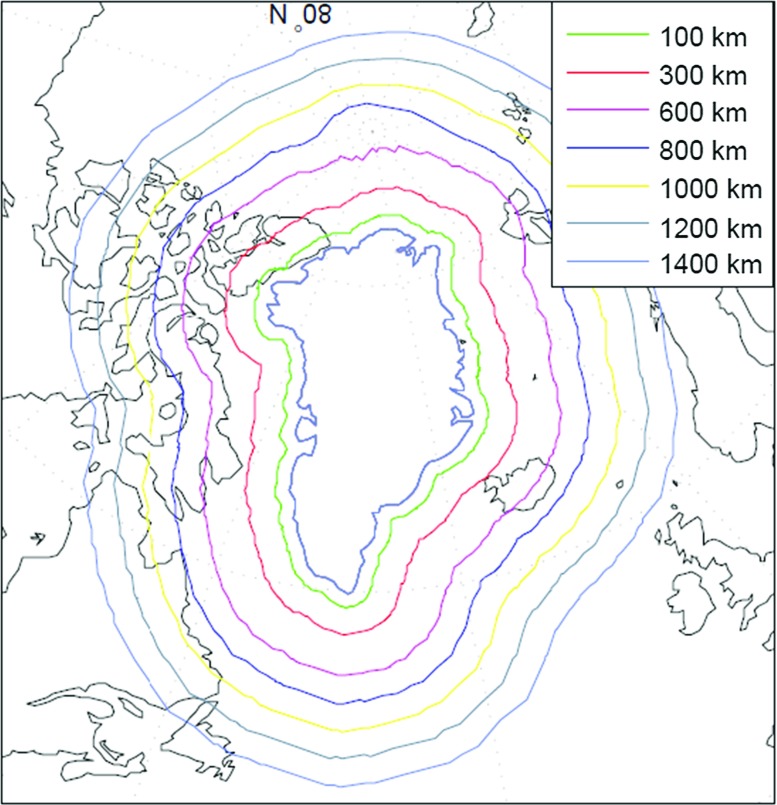
Buffer zones around Greenland considered in this study

### Choice of the optimal data processing strategy

There are a number of choices to be made when using the improved mascon approach:

- the size of the buffer zone around Greenland;
- the number of additional data points in the oceans outside the data area;
- the number of mascons covering entire Greenland;
- the choice of the least-squares estimator (i.e. ordinary least-squares versus weighted least-squares);
- the number of eigenvalues to be retained when computing an approximate inverse of the noise variance–covariance matrix $\mathbf {C_d}$.

In a series of numerical experiments, we have investigated various choices. For each choice, 100 solutions have been computed each using a different random noise realization. Other error sources were kept the same in all experiments. Each solution has been converted into mass anomalies per mascon (in Gt) and then summed up over all “Greenland” mascons to yield the total mass anomalies over entire Greenland. The total mass anomalies are then compared with the “true” ones; the RMS difference between estimated and true total mass anomalies is used as a measure of the quality of the solution.

In this way, we found the optimal choice of the various parameters mentioned before, which is shown in Table 2. In the next sections, we show how the inversion results deteriorate if a suboptimal choice is made. In each test, only one parameter is changed. Regarding data weighting, we always compute two solutions: a weighted least-squares solution (weight matrix is the inverse of the full noise covariance matrix) and an ordinary least-squares solution (weight matrix is the unit matrix).

#### Width of the buffer zone around Greenland

**Fig. 7:**
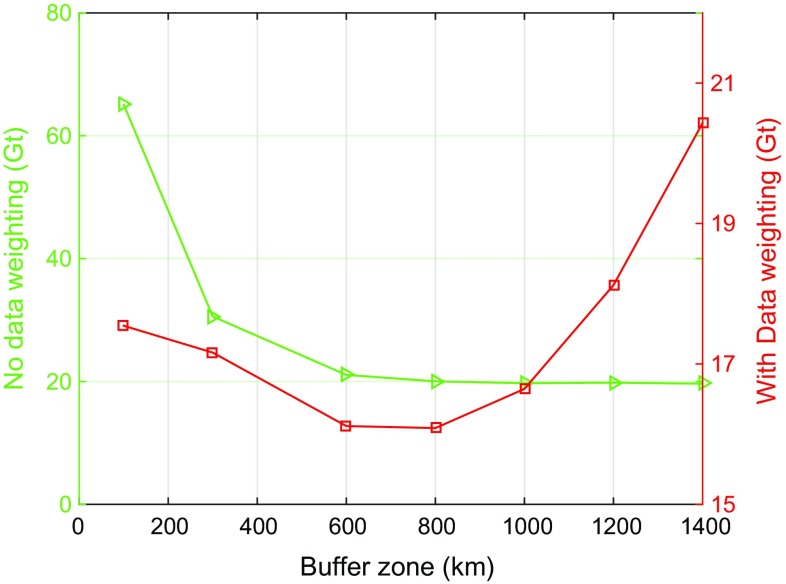
RMS error of estimated mass anomalies as a function of the buffer zone size. *Red* with data weighting, *green* without data weighting. Different vertical scales are used when plotting the *red* and *green curves*

It is well known that a buffer zone beyond the area of interest is necessary (Baur 2013). In this study, the extension is referred to as the buffer zone. To investigate the impact of the choice of the buffer zone on the estimated mass anomalies over entire Greenland, we consider buffer zones varying from 100 to 1400 km (cf. Fig. 6). For each choice of the buffer zone a weighted least-squares solution and the ordinary least-squares solution are computed. The other parameters are set equal to the values shown in Table 2. The resulting RMS error of the recovered Greenland mass anomalies is shown in Fig. 7. Using a weighted least-squares estimator, the RMS error is minimum for a 800-km buffer zone, though other choices only increase the RMS error with a few Gt. From this we conclude that when using a proper data weighting, the solution is quite robust against the choice of the buffer zone. The situation is different when an ordinary least-squares estimator is used. The smallest RMS errors are obtained for buffer zones larger than 600 km with little variations. For smaller buffer zones, however, the RMS errors increase quickly and attain values which are a few tens of Gts higher than the minimum. Overall, the RMS error of a weighted least-squares solution is always smaller than the RMS error of an ordinary least-squares solution.

#### Using data points distributed over the oceans globally

**Fig. 8:**
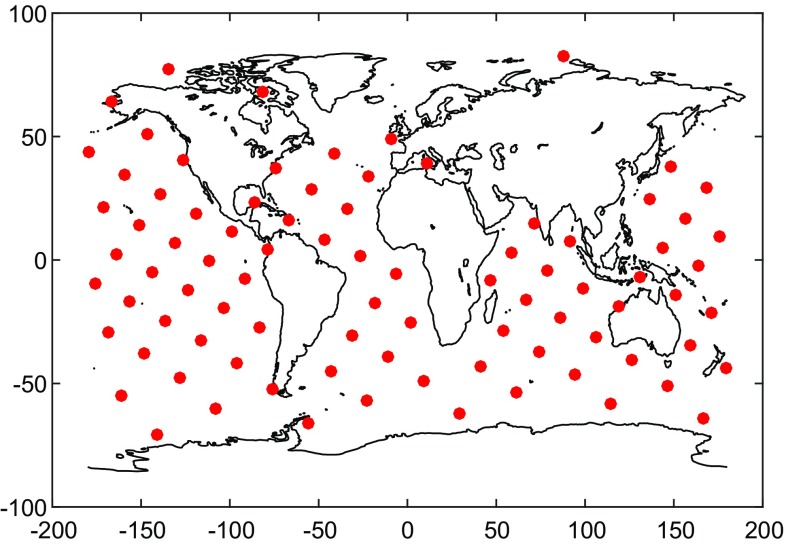
Location of additional data points over the oceans. The mean distance is about 2000 km

GRACE-based SHCs at very low degrees (particularly at degree 2) are relatively inaccurate. In principle, the implemented data weighting should suppress noise which originates from these low-degree coefficients (Chen et al. 2005). However, in regional studies as considered here, the contribution of different low-degree SHCs cannot be separated. Therefore, any attempt to suppress noise in the very low-degree SHCs may introduce a bias in the estimated mass anomalies over entire Greenland. For instance, eliminating the $C_{20}$ may reduce the estimated trend over 2003–2013 of GrIS mass variation by $\sim$18 Gts. To avoid such a bias, we add additional data points. To avoid that they capture signal below them, and they are confined to the oceans. Figure 8 shows the geographic location of these additional data points.

The additional data points are located on a Fibonacci grid with a mean distance of about 2000 km. Solutions are computed with and without the additional data points. A comparison of these solutions reveals that the added value of using additional data points is 0.02% when using ordinary least-squares and 0.5% when using weighted least-squares. Though the improvement is minor, we recommend to add additional data points in regional studies. The numerical complexity does not change much as the total number of extra points is very limited.

#### Optimal number of mascons over Greenland

**Fig. 9:**
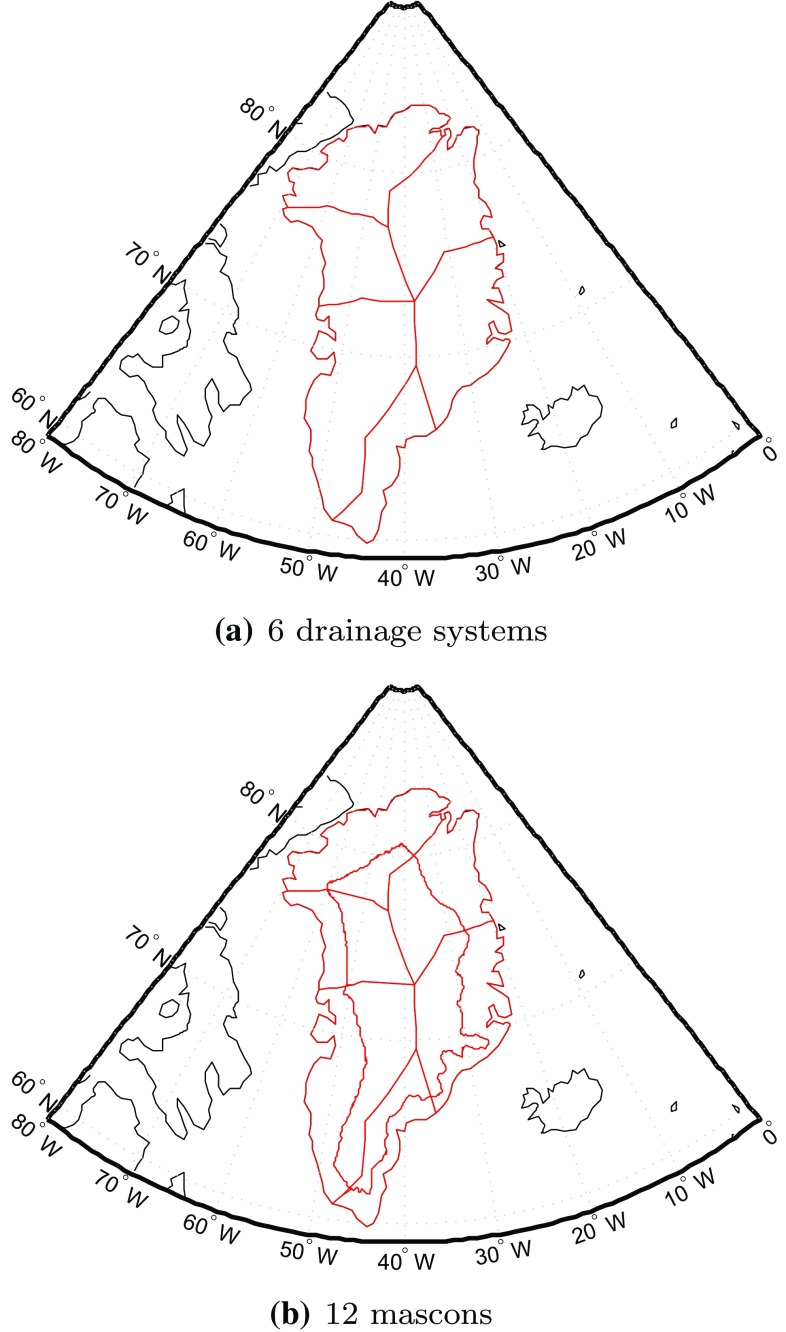
Partitioning of Greenland into 6 and 12 mascons, respectively, in line with Luthcke et al. (2006). **a** 6 drainage systems, **b** 12 mascons

**Fig. 10:**
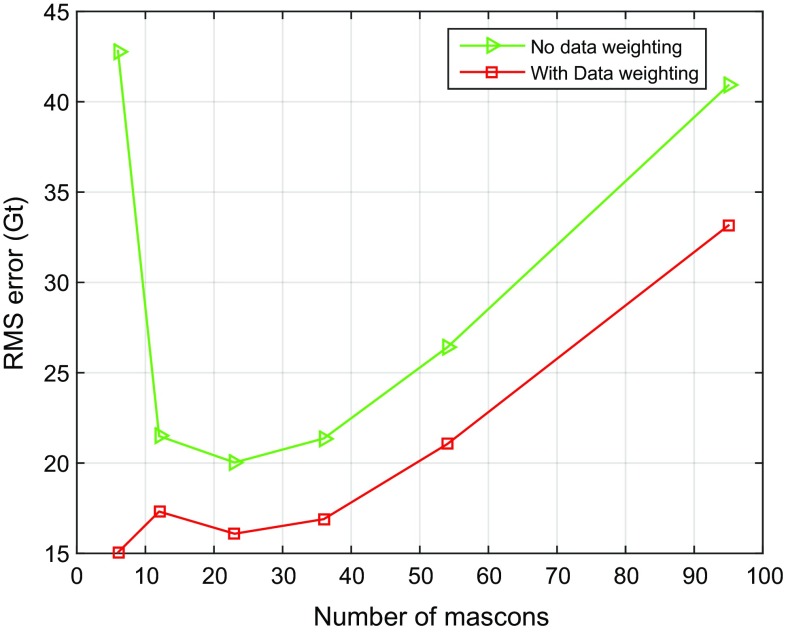
RMS errors in estimated mass anomalies over Greenland as a function of the number of mascons

In this test, we split the territory of Greenland into mascons of different sizes: from approximately $300 \times 300$ km to approximately $150 \times 150$ km, which corresponds to the number of mascons ranging from 23 to 95 (see Fig. 1). In addition, we consider also the division of Greenland into 6 or 12 mascons, as proposed in (Luthcke et al. 2006) (Fig. 9). The RMS differences between the recovered and true mass anomaly estimations are shown, as a function of the number of mascons over entire Greenland, in Fig. 10. We notice a significant reduction in the RMS error when a weighted least-squares estimator is used, between 19 and 65%, depending on the size of the mascons.

**Fig. 11:**
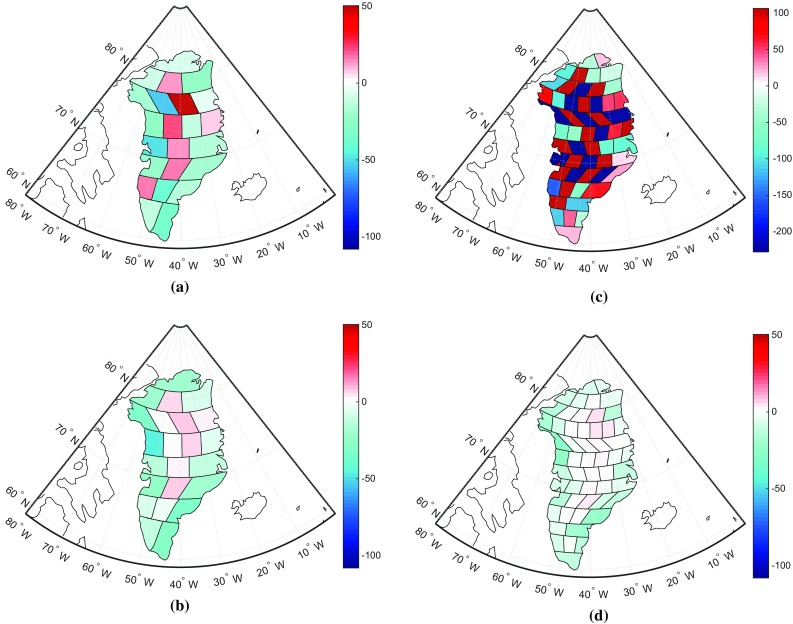
**a** Spatial pattern of recovered mass anomaly per mascon. They are estimated from the data that were contaminated by the errors presented in Figs. 4 and 5 (Gt). **b** For a better visual comparison, the true signal defined in Fig. 3 is spatially resampled to 23 mascons and shown in the unit of Gt. Similar to (**a**) and (**b**), (**c**) and (**d**) are for 54 mascons

From the green curve in Fig. 10, obtained without optimal data weighting, we find that the RMS error in the case of 6 mascons is larger than that for 23 mascons. Note that the numerical study shown in Fig. 11 considered all noise types, including random noise and representation error. It is also worth noticing that when using weighted least-squares, the quality of results based on 6 drainage systems is slightly higher than that based on 23 mascons (see the red curve in Fig. 10). This is caused by the fact that the random noise in the case of 6 mascons is reduced (i.e. from 15 to 9 Gt), as compared to 23 mascons. The numbers of 15 and 9 Gt are the result of additional numerical studies where random noise was the only error source. We did not include the details in the form of tables and figures in an attempt to limit the length of the paper and because they are not critical for the main conclusions of our study. As the difference of the RMS values in the cases of 6 and 23 mascons (see the red curve in Fig. 10) is rather small and 23 mascons provide a much better spatial resolution than 6 mascons, we recommend using 23 mascons.

The estimated mass anomalies for 23 mascons are shown in Fig. 11a; they are estimated from the data that were contaminated by the errors presented in Figs. 4 and 5. We find that in general the recovered mass anomalies show some agreement with the true signal. For instance, the mass losses take place in the coastal area and are mainly located in the north-west and south-east of Greenland. However, we could also find that the recovered mass per mascon does not exactly represent the spatial pattern of the signal. This finding is consistent with Baur (2013) and Bonin and Chambers (2013). For instance, the recovered spatial pattern in the inner part of Greenland noticeably deviates from the true signal. The recovered solution is much worse when using too many (i.e. 54) mascons as shown in Fig. 11. Due to a small size of mascons (about $150 \times 150$ km), the recovered mean mass anomalies are quite unstable, with many positive and negative estimates next to each other.

#### Number of eigenvalues retained in the approximate inversion of the noise covariance matrix

The high condition number of the noise covariance matrix does not allow a stable computation of the weight matrix, and some regularization is necessary. In this study, we use a truncated eigenvalue decomposition to improve the condition number prior to inversion (cf. Sect. 2.4). In order to estimate the optimal number of eigenvalues to be retained, we consider values between 200 and 1,600. The dimension of the noise covariance matrix is $6953 \times 6953$ in our case.

**Fig. 12:**
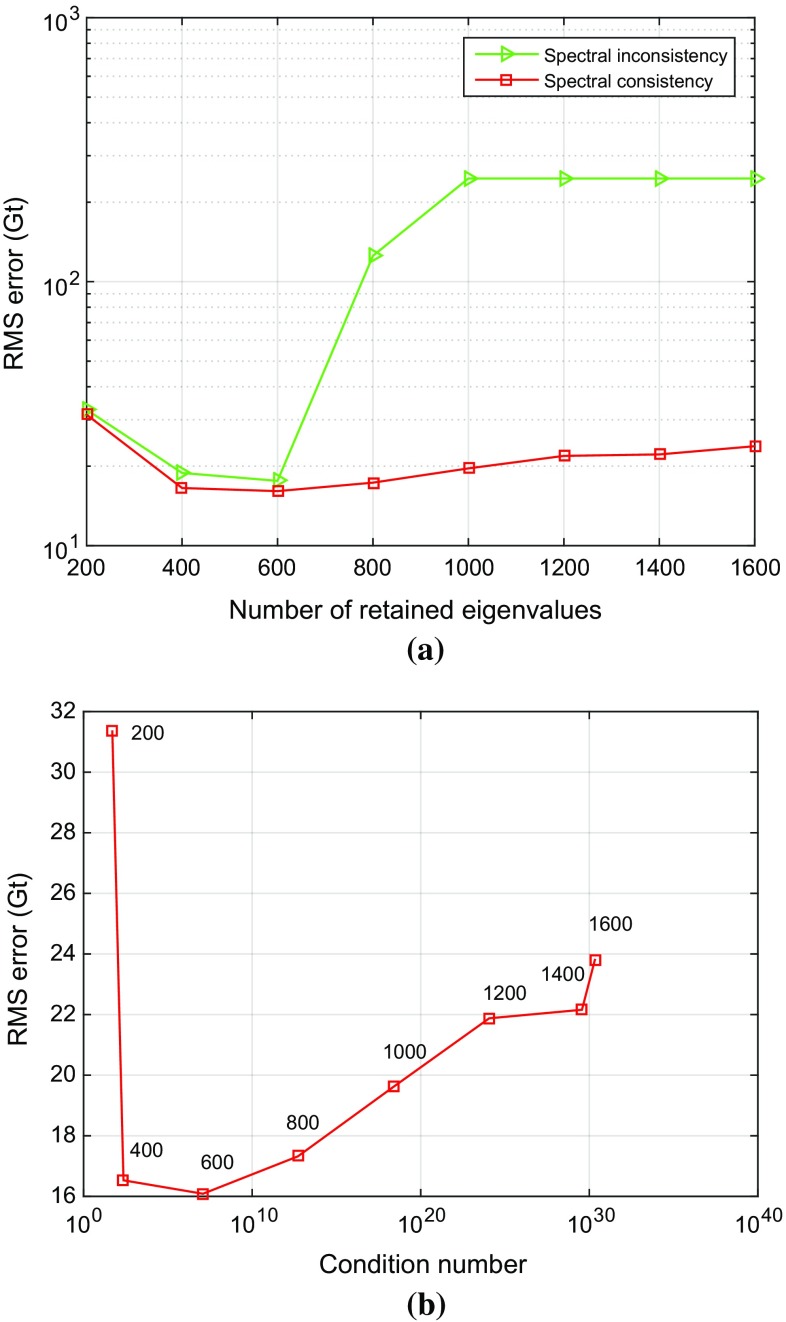
RMS errors in estimated mass anomalies as a function of **a** the number of retained eigenvalues and **b** the condition number after truncation

The RMS error of the estimated mass anomalies over Greenland is relatively large when only 200 eigenvalues are retained, but decreases by 49%, as the number of retained eigenvalues increases to 600 (see the red curve in Fig. 12a). A further increase also increases the RMS error. Therefore, we retain only the first 600 eigenvalues, i.e. about 10%. The condition number of the noise covariance matrix obtained in this way is $1.2 \cdot 10^7$. Based on Fig. 12b, which shows the same RMS error as a function of the condition number, we conclude that in general it makes sense to keep the condition number below a value of $10^7$.

### Spectral consistency

As explained in Sect. 2, the parameterization of the signal has to be spectrally consistent with the data. In this section, we demonstrate the importance of that requirement, as this requirement has not been fulfilled in previous studies. A series of tests will be done. For each test, two solutions are computed. One, which is already considered in the previous section, uses the low-pass-filtered design matrix $\mathbf {A}$, and the other one, the unfiltered design matrix, $\mathbf {A}'$ (cf. Eq. (9)). In all tests, the “true” data are generated using the design matrix $\mathbf {A}$. The number of eigenvalues which are retained in the data weighting varies between 200 and 1600.

Figure 12a shows the RMS error of the estimated total mass anomalies as a function of the retained eigenvalues. There are hardly any differences between the solutions using design matrix $\mathbf {A}'$ compared to $\mathbf {A}$ if no more than 600 eigenvalues are retained. Above 600 eigenvalues, the RMS error increases quickly if the design matrix $\mathbf {A}'$ is used and attains values close to the signal. We explain this high RMS error with the fact that the estimated mass anomalies go to zero. When using the spectrally consistent design matrix $\mathbf {A}$, the RMS error is almost the same (around 20 Gts) if at least 400 eigenvalues are retained. From this experiment we conclude that spectral consistency is important to obtain high-quality mass anomalies.

In addition, we perform a number of experiments to demonstrate the importance of using realistic signal spectra in GRACE numerical studies in general. In those tests, the unfiltered design matrix $\mathbf {A'}$ is used not only to invert gravity disturbances, but also to simulate them on the basis of yearly mass changes (Sect. 3.1.1). In that sense, the mascon functional model in these tests is consistent with the input data. At the same time, the simulated data are not realistic in the sense that the generated signal is not band-limited unlike signals which are represented by a truncated spherical harmonic series. Furthermore, the only error source considered in these tests are random errors. Data weighting is used when estimating the mass anomalies.

**Fig. 13:**
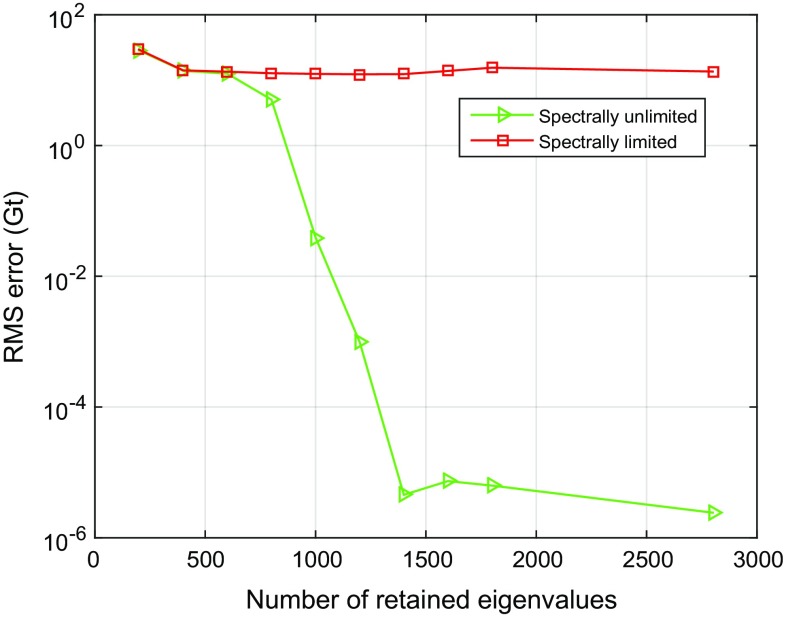
RMS errors of estimated mass anomalies over entire Greenland as a function of the number of retained eigenvalues. Data weighting is applied

**Fig. 14:**
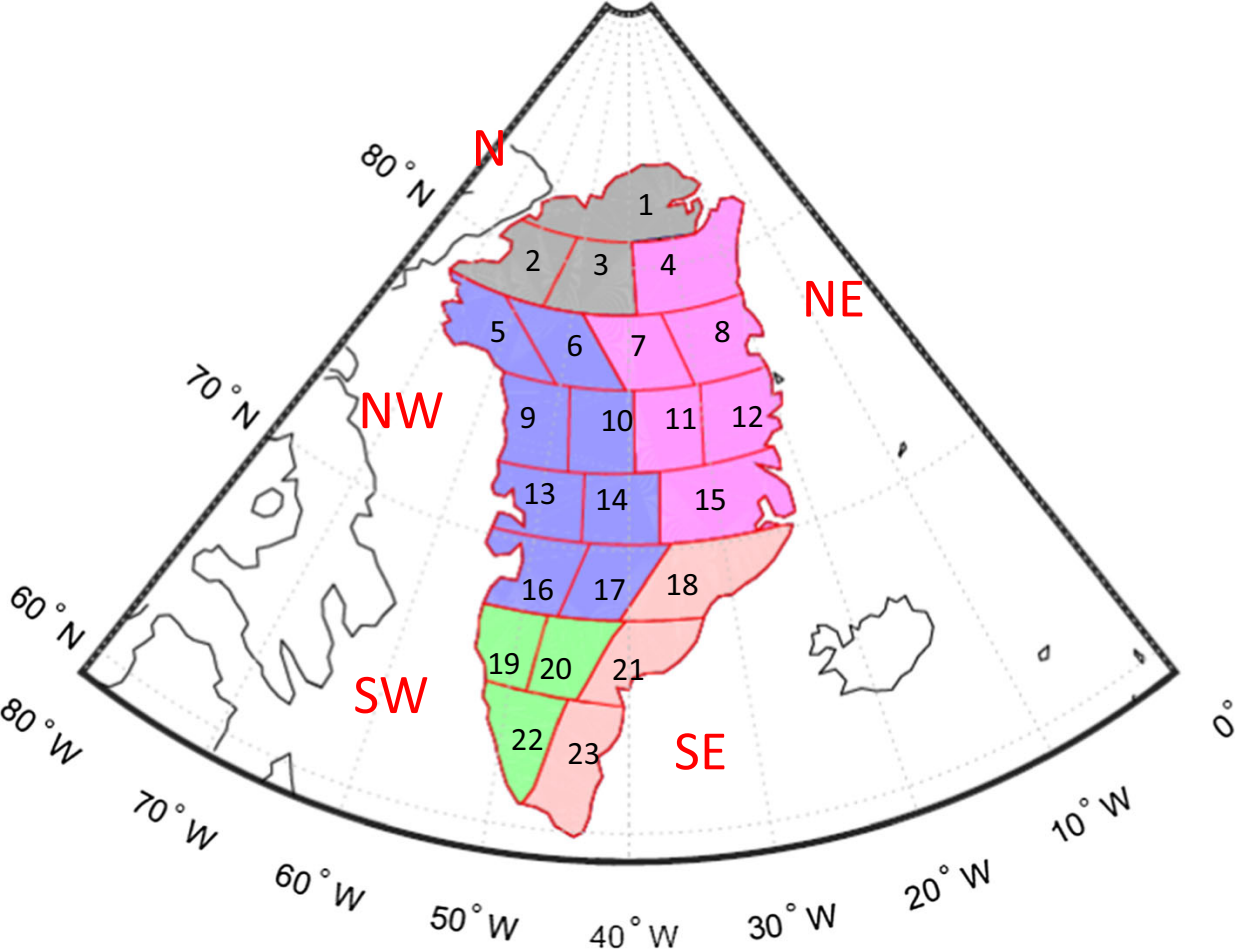
Partitioning of Greenland into 23 mascons, and the definition of the five individual drainage systems

The tests are performed for different numbers of retained eigenvalues in the spectral representation of the matrix $\mathbf {C_d}$. As shown in Fig. 13, an unrealistic (not band-limited) signal spectrum provides error estimates of the mass anomalies, which are too much small. If the number of retained eigenvalues exceeds 1400, the estimated formal RMS uncertainties of the mass anomalies are $10^{-6}$ Gt. We explain this by a spectral mismatch between signal and noise. Whereas in these experiments the signal bandwidth is not band-limited, the generated data noise is band-limited to a maximum spherical harmonic degree 120. Thus, signal above degree 120 is considered as being noise-free. Then, the exploited data inversion procedure, which suppresses data noise in the statistically optimal way, manages to exploit that high-frequency error-free signal in the recovery of mass anomalies. From these experiments, we conclude that when ignoring a proper reproduction of the signal content in numerical tests, the obtained results may be over-optimistic, particularly when a weighted least-squares estimator is used.

This experiment also explains the poor performance of the statistically optimal data inversion in the presence of spectral inconsistencies, which have been reported in the previous section. In that case, the applied data weighting assigns unrealistically high weights to high-frequency components of the signal. These signal components, however, have been removed when low-pass-filtering the design matrix. Then, the estimated mass anomalies tend to zero when more and more eigenvalues of the matrix $\mathbf {C_d}$ are retained.

## Real GRACE data analysis

The performance of the proposed approach is analysed using real GRACE data. Here we use Release-05 GRACE monthly gravity field solutions from CSR from January 2003 to December 2013. Missing months are not interpolated, but just left out. Each monthly solution is provided as a set of SHCs complete to degree 96 including a full noise covariance matrix. We replace the $C_{20}$ coefficient of all monthly solutions with estimates based on satellite laser ranging (Cheng et al. 2013). Degree-one coefficients are taken from Swenson et al. (2008) including noise variances. The Glacial Isostatic Adjustment (GIA) signal in GRACE data is removed using the model compiled by A et al. (2013).

The data are used to compute a time-series of Greenland mass anomalies. To that end, we follow the recommended data processing set-up, which is summarized in Table 2. We compute both weighted least-squares solutions and ordinary least-squares solutions.

The results are analysed in three different ways. In Sect. 4.1, we quantify the noise in the time-series of estimated Greenland mass anomalies using only the data themselves. The method applied is briefly described in Sect. 4.1. In Sect. 4.2, we compare the GRACE-based time-series (after correction for ice discharge) with time-series of SMB synthesized from the RACMO 2.3 model. We evaluate mass anomalies not only for entire Greenland, but also for individual drainage systems. In line with van den Broeke et al. (2009), we merge the 23 patches into five drainage systems: North (N), Northwest (NW), Southwest (SW), Southeast (SE) and Northeast (NE), cf. Fig. 14. In Sect. 4.3, a comparison between the estimates in this study and other mascons solutions is presented.

**Table 3:** VCE-based noise standard deviations (in Gt) of estimated mass anomalies for (i) entire Greenland and (ii) five individual drainage systems

Data weighting	N	NW	SW	SE	NE	GrIS
No	14	49	30	39	34	33
Yes	9	16	9	17	17	16
Reduction (%)	35	67	69	56	52	50

**Fig. 15:**
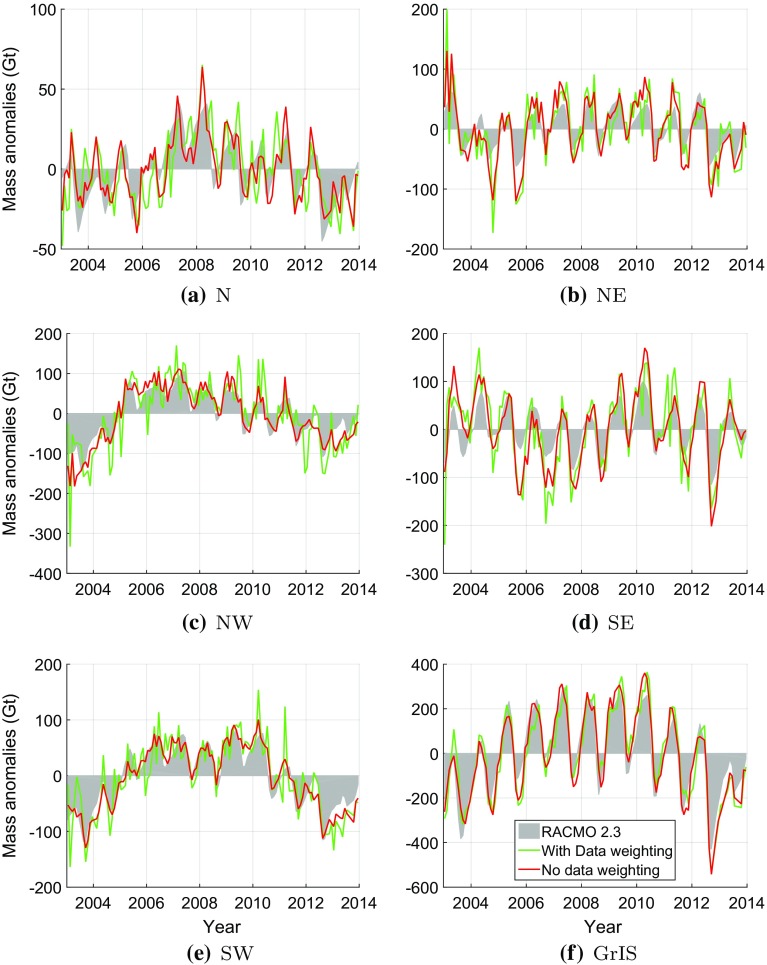
De-trended mass anomaly time-series based on modelled SMB (outlined by the *grey zone*) and GRACE data, for individual drainage systems and entire Greenland. GRACE-based time-series were computed with (*red*) and without (*green*) data weighting

**Fig. 16:**
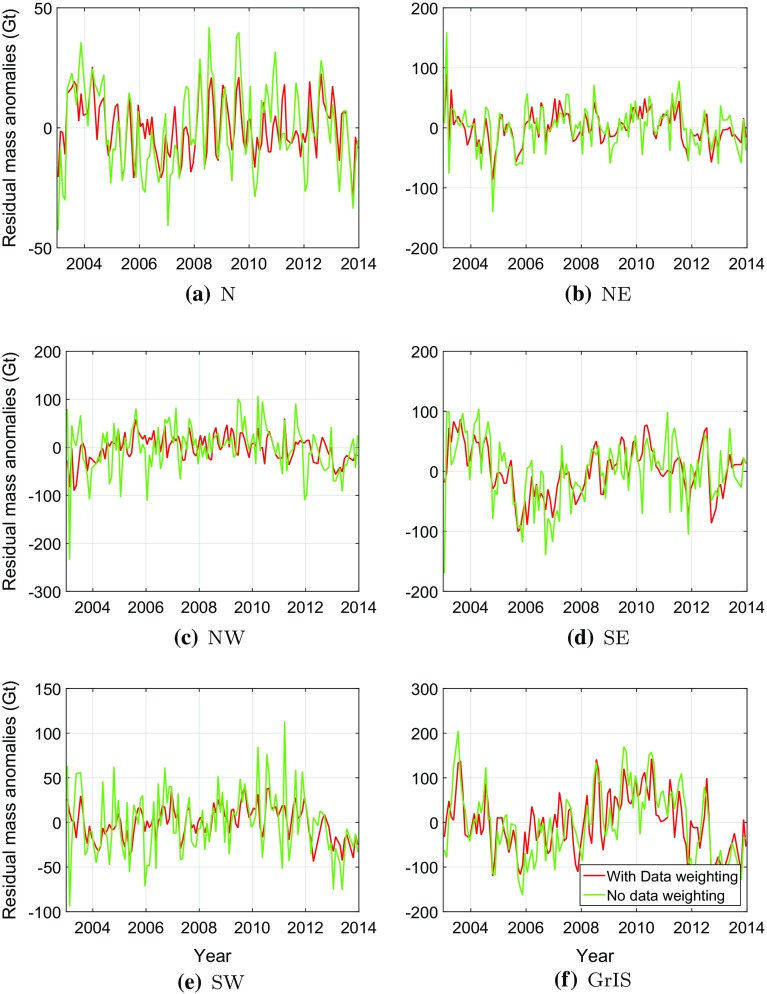
Differences of SMB-based and GRACE-based de-trended mass anomaly time-series for individual drainage systems and entire Greenland. GRACE-based time-series were computed with (*red*) and without (*green*) data weighting. **a** N, **b** NE, **c** NW, **d** SE, **e** SW, **f** GrIS

### Estimating mass anomaly uncertainties

First of all, we quantify noise in mass anomaly time-series using an original approach that does not require any independent reference. The approach is based on the assumptions that (i) true signal in the data time-series is close (but not necessarily equal) to a combination of an annual periodic signal and a linear trend; (ii) noise in the data time-series is uncorrelated and (optionally) non-stationary; and (iii) time-series of noise variances is known up to a constant scaling factor. The basic idea is to approximate the original data time-series $\mathbf {d}$ with a regularized one $\mathbf {x}$, for which purpose the following functional is minimized:

23 $$\begin{aligned} {\varPhi }[\mathbf {x}] = \frac{1}{\sigma _d^2}\left( \mathbf {d} - \mathbf {x}\right) ^\mathrm{T} \mathbf {P} \left( \mathbf {d} - \mathbf {x}\right) + \frac{1}{\sigma _x^2} \mathbf {x}^\mathrm{T} \mathbf {R} \mathbf {x}, \end{aligned}$$

where $\mathbf {P}$ is a diagonal weight matrix, which accounts for temporal variations of noise level; $\sigma _d^2$ is the noise variance; $\sigma _x^2$ is the signal variance; and *R* is a regularization matrix, which is designed such that periodic annual signals and a linear trend in the data are not penalized. An estimation of the noise variance $\sigma _d^2$ and the signal variance $\sigma _x^2$ is a part of the regularization procedure. To that end, the variance component estimation (VCE) technique (Koch and Kusche 2002) is used. This technique is iterative: at each iteration, the estimates of $\sigma _d^2$ and $\sigma _x^2$ are updated, which allows for a better regularization of the original time-series and, therefore, in a better estimation of $\sigma _d^2$ and $\sigma _x^2$ at the next iteration. As soon as the procedure converges, the latest estimate of $\sigma _d$ is used as the measure of standard deviation of random noise in the considered data. A more extended presentation of this approach will be the subject of a separate publication.

In this study, we use this approach to quantify the uncertainties in mass anomaly estimates both for entire Greenland and for the five drainage systems mentioned before.

Table 3 summarizes the main results. They confirm that, compared to an ordinary least-squares solution, optimal data weighting reduces random noise in mass anomaly estimates substantially. The largest reduction, 69%, is observed for the SW drainage system. This is likely due to a relatively large contribution of random noise to the estimated mascon of this drainage system, so that the statistically optimal data weighting becomes particularly efficient. An increased level of random noise over the SW drainage system can be explained by its relatively small size. The smallest reduction in random noise, which is observed in the NE drainage system, is still substantial, about 35%. For entire Greenland, the random noise is reduced by a factor of two.

### Validation against modelled SMB time-series

The estimated mass anomalies are compared with modelled SMB estimates over the period 2003–2013 computed using the Regional Atmospheric Climate Model (RACMO) version 2.3 (Noël et al. 2015). The spatial resolution of the RACMO 2.3 model is 11 $\times$ 11 km (see Table 1). We integrate the daily SMB estimates over time to produce daily mass anomalies, and then compute on their basis monthly mean values, to be consistent with the temporal resolution of GRACE. Finally, the computed mass anomalies are spatially integrated over individual drainage systems and over entire Greenland, respectively.

The mass anomalies derived from GRACE account for both SMB and ice discharge. According to van den Broeke et al. (2009), ice discharge manifests itself mostly as a long-term trend, whereas the seasonal mass variations are largely attributed to surface processes. In view of that, we de-trend both SMB- and GRACE-based time-series prior to their comparison. To that end, we approximate each of them with the analytic function *f*(*t*):

24 $$\begin{aligned} \begin{aligned} f(t)&=A+B(t-t_0)+ C\sin \omega (t-t_0) + D\cos \omega (t-t_0)\\&+E\sin 2\omega (t-t_0)+ F \cos 2 \omega (t-t_0), \end{aligned} \end{aligned}$$

where *A* to *F* are constant coefficients, which are estimated using ordinary least-squares, $t_0$ is the reference epoch defined as the middle of considered time interval, and $\omega = \frac{2\pi }{T}$ with $T = 1$ year. The de-trending comprises the first two terms of *f*(*t*). After de-trending, the residual GRACE-based and SMB-based time-series are compared. In the comparison, GRACE-based mass anomalies produced both with and without data weighting are considered. The de-trended GRACE-based and SMB-based time-series are shown in Fig 15 with and without using data weighting. Remarkable is the erratic behaviour of GRACE-based time-series per drainage system when no data weighting is used. This erratic behaviour is averaged out when computing mass anomaly time-series for entire Greenland.

Figure 16 shows the time-series of the differences between GRACE-based and SMB-based time-series of mass anomalies. Statistics of the differences are shown in Table 4. When data weighting is used, the differences are much smaller compared to solutions without data weighting. The most significant improvement is attained in the SW drainage system, which is consistent with the results obtained with the VCE technique (cf. Sect. 4.1). At the same time, the improvement observed for entire Greenland is smaller, about 17%, than those of individual drainage systems (24–47%). This is likely due to the fact that when summing up mass anomalies per mascon to get the mass anomalies of entire Greenland, the random noise is reduced by averaging out. Therefore, a relatively low level of random noise can be achieved for the estimates of entire Greenland, compared with the estimates per mascon. However, this will not affect the determination of other optimal parameters in Table 2. Because our operation (i.e. summing up mass anomalies per mascon to get the mass anomalies of entire Greenland) is applied to the final estimates. As a result, the remaining difference in Fig. 16f should rather be explained by residual physical signals than by noise. Such signals may reflect nonlinear mass variations not related to SMB, such as inter-annual variability in ice discharge or meltwater retention. A physical interpretation of these signals is outside the scope of this study.

### Comparison with Greenland mass anomalies from other studies

**Fig. 17:**
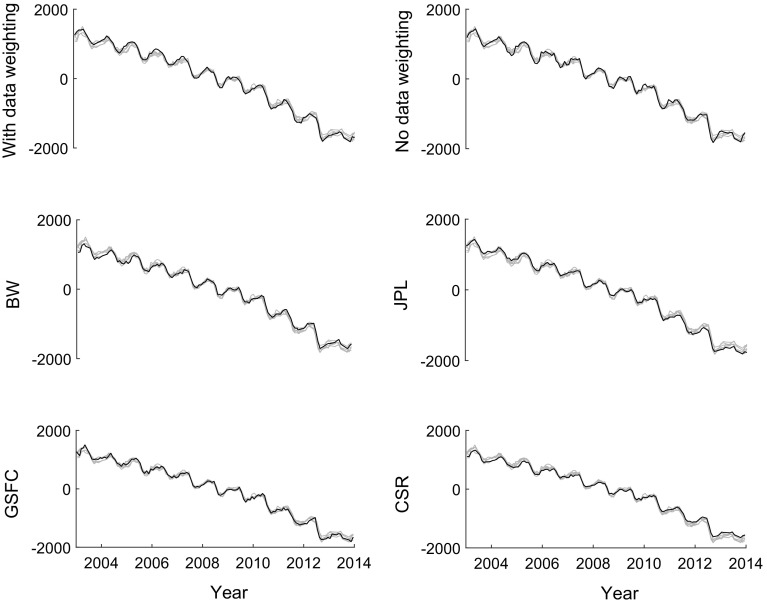
Mass anomaly time-series produced in this study with data weighting and without data weighting, as well as by Wouters et al. (2008) (marked as “BW”), JPL, GSFC and CSR. The unit of *Y*-axis is mass variation in Gts. Each *plot* highlights only one solution (*black line),* whereas other solutions are shown in *grey*

The mass anomaly estimates are further compared with those based on existing global and regional mascon solutions, as well as with results from the literature. The available global mascon solutions discussed in this study are the products released by JPL (Watkins et al. 2015), GSFC (Luthcke et al. 2013) and CSR (Save et al. 2016). Note that these mascon solutions are estimated from GRACE KBR data, while the method developed in this study uses GRACE SHCs. We also include the regional mascon solution by Wouters et al. (2008), which also takes GRACE SHCs as input. As shown in Fig. 17, different mass anomaly time-series of entire Greenland agree with each other very well. The same applies to the linear trend estimates, which are shown in Table 5.

**Table 4:** Ice discharge-corrected RMS differences (in Gts) between GRACE-based mass anomaly estimates and SMB-based mass anomalies for (i) entire Greenland and (ii) five individual drainage systems

Data weighting	N	NW	SW	SE	NE	GrIS
No	16	48	34	54	37	76
Yes	12	27	18	41	27	63
Reduction (%)	28	44	47	24	27	17

As before, we use VCE-based estimation of random noise standard deviations and a validation against modelled SMB estimates to assess the quality of the various mascon solutions. The smallest noise standard deviation (16 Gt) is observed for the solution produced in this study with the optimal data weighting (Table 6). A comparable noise standard deviation (19 Gt) is estimated for the JPL solution, whereas standard deviations for other solutions are much larger. When validating against independent SMB output, the solution produced in this study with the optimal data weighting shows, again, the best performance (see Table 6). From Table 6, it follows that relatively low VCE-based standard deviations in the JPL solutions do not indicate a better quality. This might be caused by the fact that the application of spatio-temporal constraints in the production of those solutions could reduce random noise, but at a price of making the estimates biased towards a priori information, which is reflected in the applied constraints. The bias becomes visible when validating with independent data such as SMB model estimates. This justifies our decision not to apply any spatial or temporal constraints in producing our solutions in order to minimize biases. A rapid mass loss in an area of a limited size is a particularly challenging scenario for any method for mass anomaly estimation. In particular, the impact of a bias caused by the applied constraints can be particularly large in that case. An in-depth discussion of this issue is beyond the scope of this manuscript. A further discussion of biases introduced into mascons solutions by various spatio-temporal constraints will be the subject of a separate article.

## Summary and conclusions

In this study, we proposed an improved mascon approach compared to the previous studies of Forsberg and Reeh (2007) and Baur and Sneeuw (2011). Based on numerical experiments, we optimize various parameters shown in Table 2. The proposed methodology allows the estimation of mass anomalies over Greenland in a statistically optimal way, by propagating the full noise covariance matrices of SHCs into full noise covariance matrices of gravity disturbances at altitude, which are then used as data in the mass anomaly estimation scheme. We show that the data weighting improves the accuracy of the estimated mass anomalies substantially. The high condition number of the noise covariance matrix is addressed successfully using a truncated eigenvalue decomposition, which retains about 10% of the eigenvalues corresponding to a condition number of about $10^7$. We also demonstrated that the optimal size of a mascon is about $300 \times 300$ km, which implies about 23 mascons for Greenland. This finding is consistent with the spatial resolution of GRACE reported in the literature (Longuevergne et al. 2010; Ramillien et al. 2004; Beighley et al. 2011). Furthermore, we have proven that spectral consistency of the mass anomaly model and the data is very important to obtain accurate estimates of the mass anomalies. If data weighting is applied, a spectral inconsistency makes the recovery of mass anomalies non-robust and provides severely biased estimates. This is more pronounced if more eigenvalues of the noise covariance matrix are retained. Then, the high-frequency components of the model are over-weighted, resulting in gravity anomalies close to zero, because high-frequency signal is absent in the data. The maximum degree in the low-pass filter applied to maintain a spectral consistency must be consistent with the GRACE solutions utilized to generate the pseudo-observations. More specifically, in the simulation, we choose the maximum degree to be 120, in line with the DMT solutions. However, in the real data processing, the CSR solutions are utilized. Then, the maximum degree is 96, in line with the CSR solutions.

**Table 5:** Greenland mass anomaly trends over the period 2003–2013 (in Gt/year) estimated from different solutions and experimental set-ups

Different estimates	Trend
With data weighting (this study)	$-$286
No data weighting (this study)	$-$276
JPL mascon	$-$289
CSR mascon	$-$262
GSFC mascon	$-$283
Wouters et al. (2008)	$-$264
Velicogna et al. (2014)	$-$280
Schrama et al. (2014)	$-$278

**Table 6:** VCE-based noise standard deviations (in Gts) of estimated mass anomalies (left column) and ice discharge-corrected RMS differences (in Gts) between GRACE-based mass anomaly estimates from different mascon solutions and SMB-based mass anomalies (right column). All the estimates refer to entire Greenland

Different estimates	VCE-based noise	Ice discharge-corrected
	standard deviations	RMS differences
With data weighting (this study)	16	63
No data weighting (this study)	33	76
JPL mascon	19	73
CSR mascon	29	70
GSFC mascon	45	76
Wouters et al. (2008)	36	79

It is worth stressing that the set of parameters shown in Table 2 is optimal if the main goal is to estimate mass anomalies over a one-year interval. This scenario represents a kind of intermediate choice between the two extremes of a monthly signal and a mean signal over a multi-year time interval (e.g. a long-term linear trend). In our latest studies, we found that the optimal data processing scenario definitely depends on the temporal scale of interest. If, for instance, the main focus is on a long-term trend, the impact of random noise (north–south stripes) is minor, so that other types of noise (particularly the parameterization error) become dominant. In that case, the way to improve the quality of the estimates is reducing the size of individual mascons and applying a data weighting based on provided error covariance matrices of GRACE monthly solutions. On the other hand, if the main research interest is month-to-month mass anomaly variations, random noise by far exceeds noise of other types, including the parameterization errors. Then, the best results are obtained when the size of individual mascons is relatively large, whereas the data weighting based on provided error covariance matrices is switched on. These and other findings are discussed in detail in a separate manuscript.

We also applied the proposed data processing scheme to real GRACE data and computed mass anomaly time-series for five drainage systems and entire Greenland. Using VCE, we found that when a proper data weighting is used, the accuracy of the estimated mass anomalies increases by a factor of 1.5 to 3.0, depending on the drainage system. A comparison of the GRACE-based mass anomalies with modelled SMB mass anomalies revealed that a proper data weighting provides a better fit of GRACE-based and SMB-based mass anomalies, with improvements between 24 and 47% depending on the drainage system. We consider this as indication that a proper data weighting provides much more accurate estimates of mass anomalies. The improvement is, however, marginal for entire Greenland. This is likely due to a relatively minor role of random noise when estimating mass anomalies over very large areas.

## Figures and Tables

**Fig. 18:**
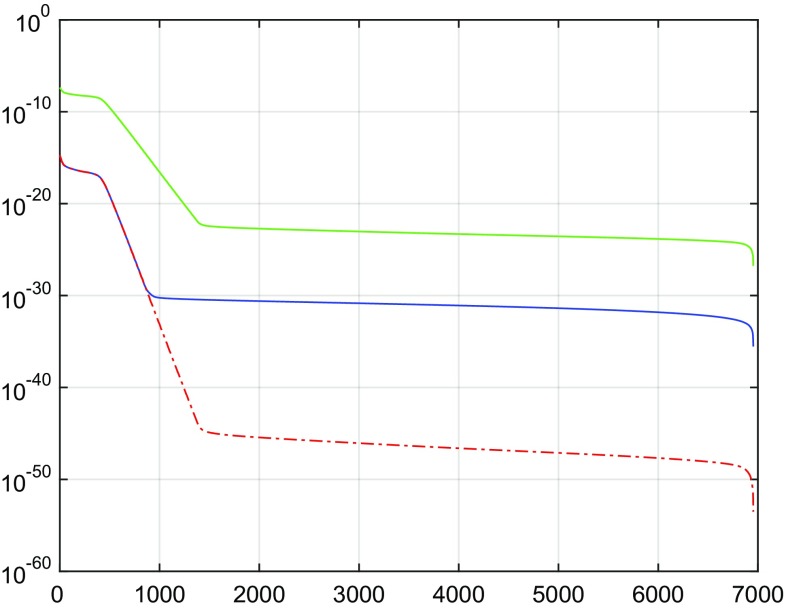
Eigenvalues of the matrix $\mathbf {C_d}$ computed directly (*blue*) and using the procedure suggested in this study (*red*). The singular values of the matrix $\mathbf {H}$ are shown in green

## Appendix A. Eigenvalue decomposition of the noise covariance matrix $\mathbf {C_d}$

A statistically optimal inversion of gravity disturbances into mass anomalies per mascon requires the inversion of the noise covariance matrix $\mathbf {C_d}$. Since this matrix is ill-conditioned some type of regularization is needed. Here, we use an eigenvalue decomposition

25 $$\begin{aligned} \mathbf {C_d} = \mathbf {Q}\varvec{\Lambda } \mathbf {Q}^\mathrm{T}. \end{aligned}$$

To minimize the loss of significant digits during the computations, we do not compute explicitly the noise covariance matrix, but apply the following procedure.

We start with Eq. (2) in matrix-vector form:

26 $$\begin{aligned} \mathbf {d}=\mathbf {F}\delta \mathbf {p}, \end{aligned}$$

where the vector $\mathbf {\delta p}$ comprises the SHCs of a monthly GRACE solution (${\varDelta } C_{lm}$, ${\varDelta } S_{lm}$) and $\mathbf {F}$ is the matrix of spherical harmonic synthesis that maps SHCs into gravity disturbances. If the noise covariance matrix of the SHCs is $\mathbf {C_{\delta _p}}$ and no constraints are applied when estimating the SHCs,

27 $$\begin{aligned} \mathbf {C_{\delta _p}}=\mathbf {N}^{-1}, \end{aligned}$$

where $\mathbf {N}$ is the normal matrix exploited in the computation of SHCs from GRACE level-1b data. The Cholesky decomposition of this matrix is:

28 $$\begin{aligned} \mathbf {N}=\mathbf {L}\mathbf {L}^\mathrm{T}. \end{aligned}$$

According to the law of covariance propagation, the noise covariance matrices $\mathbf {C_{\delta _p}}$ and $\mathbf {C_d}$ are related to each other as

29 $$\begin{aligned} \mathbf {C_d}=\mathbf {F} \mathbf {C_{\delta _p}} \mathbf {F}^\mathrm{T}. \end{aligned}$$

Substitution of Eqs. (27) and (28) into Eq. (29) gives

30 $$\begin{aligned} \mathbf {C_d}=\mathbf {F} \, \left( \mathbf {L}\mathbf {L}^\mathrm{T}\right) ^{-1} \, \mathbf {F}^\mathrm{T} = \mathbf {F} \, \left( \mathbf {L}^{-1}\right) ^\mathrm{T} \, \mathbf {L}^{-1} \, \mathbf {F}^\mathrm{T} = \mathbf {H} \, \mathbf {H}^\mathrm{T}, \end{aligned}$$

where

31 $$\begin{aligned} \mathbf {H} = \mathbf {F} \, \left( \mathbf {L}^{-1}\right) ^\mathrm{T}. \end{aligned}$$

Let

32 $$\begin{aligned} \mathbf {H}=\mathbf {U}\varvec{\Sigma } \mathbf {V}^\mathrm{T} \end{aligned}$$

be the SVD of the matrix $\mathbf {H}$, where $\varvec{\Sigma }$ is the matrix of singular values and $\mathbf {U}$ and $\mathbf {V}$ are the matrices of left and right singular vectors, respectively. Equation (32) and the equality

33 $$\begin{aligned} \mathbf {V}^\mathrm{T} \mathbf {V}=\mathbf {I} \end{aligned}$$

allow Eq. (30) to be rewritten as

34 $$\begin{aligned} \mathbf {C_d} = \mathbf {U}\varvec{\Sigma } \mathbf {V}^\mathrm{T} \mathbf {V}\varvec{\Sigma }^\mathrm{T} \mathbf {U}^\mathrm{T} = \mathbf {U}\varvec{\Sigma } \varvec{\Sigma }^\mathrm{T} \mathbf {U}^\mathrm{T}. \end{aligned}$$

It is easy to see that $\varvec{\Sigma } \varvec{\Sigma }^\mathrm{T}$ is a square diagonal matrix with elements $\lambda _i$ defined as

35 $$\begin{aligned} \lambda _i = \sigma _i^2 \qquad (i=1,\ldots N_d), \end{aligned}$$

where $\sigma _i$ are the singular values forming the matrix $\varvec{\Sigma }$ and $N_d$ is the number of data points. Therefore, the representation of matrix $\mathbf {C_d}$ given by Eq. (34) satisfies the properties of the eigendecomposition, so that $\lambda _i$ are the eigenvalues of $\mathbf {C_d}$,

36 $$\begin{aligned} \mathbf {Q}=\mathbf {U}, \qquad \text{ and } \qquad \varvec{\Lambda }=\varvec{\Sigma } \varvec{\Sigma }^\mathrm{T}. \end{aligned}$$

Thus, the operations prescribed by Eqs. (28), (31), (32) and (36) provide the eigenvalue decomposition of the matrix $\mathbf {C_d}$ without the need to compute this matrix explicitly.

In order to demonstrate the superior stability of the proposed computational procedure, we perform the following experiment. We use the normal equation matrix for the monthly GRACE solution of August 2006 from DMT. We compute explicitly the noise covariance matrix $\mathbf {C}_d$ and perform an eigenvalue decomposition of this matrix. Alternatively, we follow the procedure outlined before. Figure 18 shows the eigenvalues of $\mathbf {C}_d$ for both procedures. The direct computation of the eigenvalues of $\mathbf {C}_d$ provides only the first 900 eigenvalues. The flattening of the eigenvalue spectrum beyond an index of about 900 is caused by numerical round-off errors and is at the level of the largest eigenvalue times machine epsilon for IEEE double-precision arithmetic. Using the proposed procedure allows to compute the first 1400 eigenvalues before numerical round-off errors become dominant. From this we conclude that the proposed procedure is numerically more stable and, therefore, better suited to deal with ill-conditioned noise covariance matrices when computing a weighted least-squares solution.
